# Spinal anesthesia for cesarean section in a super morbidly obese parturient: A case report

**DOI:** 10.1097/MD.0000000000021435

**Published:** 2020-07-31

**Authors:** Ana Cho, Jinyoung So, Eun Young Ko, Dasom Choi

**Affiliations:** Department of Anesthesia & Pain Medicine, Soonchunhyang University Bucheon Hospital, Bucheon, Gyeonggi-do, Republic of Korea.

**Keywords:** cesarean section, morbid obesity, spinal anesthesia

## Abstract

**Introduction:**

The population of obese individuals is increasing worldwide, and as a result, the number of mothers with super morbid obesity undergoing cesarean sections is also increasing. However, little is known about which anesthetic technique is appropriate for cesarean sections of super morbidly obese parturients.

**Patient Concerns:**

A 35-year-old woman with body mass index 61.3 kg/m^2^ at a gestational age of 37 weeks.

**Diagnosis:**

The patient was super morbidly obese parturient.

**Interventions:**

Spinal anesthesia was performed. A spinal needle was inserted into the L4–5 interspinous space in the sitting position. After confirmation of cerebrospinal fluid, 0.5% hyperbaric bupivacaine 9 mg and fentanyl 20 μg were injected into the subarachnoid space.

**Outcomes:**

After the administration of spinal anesthetics, the nerve block to the T8 dermatome level was confirmed, surgery was performed, and the fetus was delivered. The patient's vital signs were stable until the end of the operation.

**Conclusion:**

There is no established strategy for selecting a method of anesthesia in patients with morbid obesity (body mass index 40 kg/m^2^ or more). For this reason and considering the amount of bupivacaine used for spinal anesthesia, we wanted to share our experience with spinal anesthesia for cesarean section in a super morbidly obese parturients.

## 1 Introductions

The population of obese individuals is increasing worldwide.^[1]^ The World Health Organization uses the body mass index (BMI) to define a normal weight as 18.9 to 24.9 kg/m^2^, overweight as 25 to 29.9 kg/m^2^, and obese as 30 kg/m^2^ or more. Class I obesity is defined as BMI 30 to 34.9 kg/m^2^, class II obesity as BMI 35 to 39.9 kg/m^2^, and class III obesity as BMI 40 kg/m^2^ and above.^[2]^ BMI 40 kg/m^2^ and above is defined as morbid obesity, and BMI 50 kg/m^2^ and above is defined as super morbid obesity.^[3,4]^ As the number of obese people increases, the number of morbidly obese parturients who undergo cesarean sections is also increasing.

In patients with morbid obesity, the frequency of a cesarean section is more than double that in normal parturients, and the risk of gestational hypertension, preeclampsia, gestational diabetes, macrosomia, and wound complications is also increased. In addition, there is a high incidence of endotracheal intubation failure during general anesthesia, a high risk of death from sequelae of the lung or heart, and a high incidence of failure and sequelae in regional anesthesia.^[5–7]^ In pregnancy, functional residual capacity decreases and oxygen consumption increases, especially in obese parturients. Therefore, maternal obesity is an important risk factor in pregnancy, and problems such as difficulty in endotracheal intubation, pulmonary aspiration, and hypoxia may occur during general anesthesia. If there is no contraindication to it, regional anesthesia is preferred to general anesthesia.

Although the number of morbidly obese parturients undergoing cesarean section is increasing, little is known about which anesthetic technique is appropriate for them. We report a case of spinal anesthesia performed during the cesarean section of a 37 weeks pregnant woman with super morbid obesity (BMI 61.3 kg/m^2^). The patient consented to the publication of this case report.

## 2 Case report

A 35-year-old woman (height 1.682 m, body weight 173.4 kg, BMI 61.3 kg/m^2^) visited our hospital for selective cesarean section at a gestational age of 37 weeks. Three years previously, the patient was diagnosed with diabetes, which was controlled with insulin. Preoperative obstetric ultrasonography confirmed normal amniotic fluid amounts and transverse presentation of the fetus. Vital signs at the time of admission were as follows: blood pressure 145/90 mm Hg, heart rate 92 beats/min, respiratory rate 20 breaths/min, and body temperature 36.8°C. The chest radiograph showed no active lesion in the lung, and electrocardiography showed a normal sinus rhythm. The results of arterial blood gas analysis, complete blood count, coagulation test, urine test, and liver function test were in the normal range. In preoperative anesthetic evaluation, spinal anesthesia was selected because of the high possibility of endotracheal intubation failure due to super morbid obesity.

The patient entered the operating room after an intramuscular injection of 0.2 mg of glycopyrrolate after 8 hours of fasting. The vital signs measured before induction of anesthesia were as follows: blood pressure 155/78 mm Hg, heart rate 84 beats/min, body temperature 37.0°C, respiratory rate 18 breaths/min, and pulse oxygen saturation level 97%. Considering the possibility of hypotension, an arterial cannula was placed in the right radial artery for invasive arterial pressure monitoring during spinal anesthesia. The patient was placed in a sitting position for easy confirmation of landmarks, and the anesthesiologist explored the surface anatomy. The L3–4 and L4–5 interspinous spaces were palpable. After sterile preparation, the skin was locally anesthetized with 20 mg of 2% liodcaine, and spinal anesthesia was performed through the L3–4 interspinous space using a 89 mm 24-gauge spinal needle. After dura puncture, cerebrospinal fluid was confirmed, and 9 mg of 0.5% hyperbaric bupivacaine and 20 μg of fentanyl were injected into the subarachnoid space. The patient was then placed in a supine position, and the block level of spinal anesthesia was checked. A sensory block test to cold alcohol pads confirmed nerve block to the T8 dermatome level. Immediately after spinal anesthesia, invasive arterial blood pressure was measured as 156/85 mm Hg and heart rate as 99 beats/min. Seven minutes after the operation began, a boy weighing 4.170 kg was delivered; the baby had Apgar scores of 7 and 9 at 1 and 5 minutes, respectively. Invasive arterial blood pressure was maintained between 165/85 and 94/38 mm Hg and heart rate was maintained between 69 and 105 beats/min. The surgery was completed uneventfully with an estimated blood loss of 600 mL. The intravenous fluids injected intravenously during surgery were 500 mL of colloid and 150 mL of crystalloid. The total operation time was 68 minutes. The block level of spinal anesthesia was maintained at the T8 dermatome level during the operation and was also confirmed at the end of operation. The patient did not experience intraoperative pain. At the end of surgery, the blood glucose level was 95 mg/dL. After confirming normal vital signs, the mother was transferred to the recovery room, and vital signs in the recovery room were normal. The patient was discharged on postoperative day 5.

## 3 Discussion

Obesity in the general population is increasing rapidly in developed countries. Consequently, the incidence of obesity in the obstetric population is also increasing. In morbidly obese parturients, the incidence of cesarean sections is twice as high as in normal parturients, and the risk of gestational hypertension, preeclampsia, gestational diabetes, macrosomia, and wound complications is increased.^[8]^ In addition, the failure rate of endotracheal intubation during general anesthesia is high, and the risk of death due to pulmonary or cardiovascular complications is high,^[9]^ in regional anesthesia, the incidence of failure and sequelae is high.^[10]^

Anesthesia methods that can be applied to a parturient undergoing cesarean section include general anesthesia or regional anesthesia. It is not known which method of anesthesia is superior for obese parturients, and for super morbidly obese parturients in particular. The patient in this case chose regional anesthesia because of the possibility of difficult intubation. Failure rate of endotracheal intubation for parturients is reported to be about 8 times higher than general surgery patients.^[11]^ The most likely cause of maternal morbidity associated with anesthesia is failure of endotracheal intubation. Hood and Dewan reported an incidence of difficult intubation of 33% in morbidly obese parturients undergoing cesarean delivery who weighed >136 kg.^[12,13]^ Because of the risks of general anesthesia, regional anesthesia is preferred for cesarean sections. According to the American Society of Anesthesiologists’ Practical Guidelines for Obstetrical Anesthesia, regional anesthesia can be successfully applied for cesarean section.

Regional anesthesia is widely used in the field of obstetric anesthesia and is the most commonly used anesthesia in cesarean section. It mitigates the risk of general anesthesia and related potential complications such as failed tracheal intubation and aspiration.^[14,15]^ Regional anesthesia for cesarean section includes epidural anesthesia and spinal anesthesia, as well as continuous spinal anesthesia and combined spinal-epidural anesthesia. The most common method for cesarean section is spinal anesthesia; the advantage is that the neuraxial block is rapid and intense. When using spinal anesthesia, a small amount of local anesthetic is used, so that the fetus is less likely to be exposed to the drug, and the risk of systemic toxicity of the local anesthetic to parturients is also low. However, there are disadvantages of single dose spinal anesthesia, including precipitous hypotension, post dural puncture headache, and limited control of the sensory level.

It is difficult to identify landmarks when performing regional anesthesia in morbidly obese patients. It is also often difficult to identify the midline and to palpate spinous processes in the obese patient population.^[16]^ Stiffler et al reported difficulty in palpating landmarks in 68% of obese nonpregnant patients compared with only 5% in those with a normal BMI.^[17]^ Additionally, in obese patients, it is easier to identify landmarks in the sitting position than in the lateral position.^[3]^ As the BMI increases, the distance to the epidural space increases.^[18]^ Therefore, spinal anesthesia in obese patients is expected to require a spinal needle longer than the standard spinal needle. However, in most obese patients, spinal and epidural anesthesia has been successfully performed with standard length needles.^[19]^

Spinal anesthesia for cesarean section is generally safe for both the mother and fetus but can often result in maternal hypotension. Reducing the dose of local anesthetics for spinal anesthesia reduces the frequency of hypotension. Many studies have reported that the incidence of hypotension is also affected by the type and amount of intravenous fluids administered before anesthesia and the type and dose of the booster used.^[20–23]^ In this case, no hypotension occurred during surgery. The dose of bupivacaine needed for spinal anesthesia for cesarean section is less than that needed for non-pregnant women. However, there is little research on the dose of bupivacaine required for cesarean section of morbidly obese parturients.^[3]^ In this case, 9 mg of hyperbaric bupivacaine was injected into the subarachnoid space and the level of blockage was at the T8 dermatome. Spinal anesthesia during cesarean section provides a strong nerve block, but there is a risk of conversion to general anesthesia with prolonged surgery. In this case, the nerve block was well maintained until the end of the operation, and no additional anesthesia was needed. However, the possibility of prolongation of the operation time due to the macrosomia and difficulty in surgical procedures during the cesarean section of obese patients should be considered in terms of epidural anesthesia and combined spinal-epidural anesthesia.

Although obesity in the general population and consequently, the incidence of obesity in the obstetric population is rapidly increasing, there is no established strategy for selecting a method of anesthesia in patients with morbid obesity (BMI 40 kg/m^2^ or more). For this reason and considering the amount of bupivacaine used for spinal anesthesia, we wanted to share our experience with spinal anesthesia for cesarean section in a super morbidly obese parturients.

## Acknowledgments

This work was supported by the Soonchunhyang University Research Fund.

## Author contributions

Ana Cho was the patient's anesthesiologist, reviewed the literature and contributed to manuscript drafting; Jinoung So reviewed the literature and contributed to manuscript drafting; Eun Young Ko reviewed the literature and contributed to manuscript drafting. Dasom Choi reviewed the literature and contributed to manuscript drafting.
